# Association of fever and infections with the subsequent risk of atopic diseases in children with genetic susceptibility for T1D: Results from the TEDDY cohort study

**DOI:** 10.1111/pai.70209

**Published:** 2025-10-02

**Authors:** Tiina Palmu, Jussi Lehtonen, Helena Elding Larsson, Kristian F. Lynch, Berglind Jonsdottir, Corrado Cilio, Desmond Schatz, Marian Rewers, Heikki Hyöty, Maria Lönnrot, the TEDDY Study Group

**Affiliations:** ^1^ Department of Virology, Faculty of Medicine and Health Technology Tampere University Tampere Finland; ^2^ Department of Dermatology Tampere University Hospital, Wellbeing Services County of Pirkanmaa Tampere Finland; ^3^ Department of Clinical Sciences Malmö Lund University Lund Sweden; ^4^ Department of Pediatrics Skåne University Hospital Malmö/Lund Sweden; ^5^ Health Informatics Institute University of South Florida Tampa Florida USA; ^6^ Childrens Hospital Iceland Reykjavík Iceland; ^7^ Department of Pediatrics University of Florida Gainesville Florida USA; ^8^ Barbara Davis Center for Diabetes, Department of Pediatrics University of Colorado School of Medicine Aurora Colorado USA; ^9^ Fimlab Laboratories Wellbeing Services County of Pirkanmaa Tampere Finland

**Keywords:** allergic rhinitis, asthma, atopic eczema, fever, infections

## Abstract

**Background:**

The increased prevalence of atopic diseases may be due to lifestyle changes affecting our exposure to microbes. We investigated the association between infections, fever, and atopic diseases in a large international birth cohort setting.

**Methods:**

3842 children at genetic risk for type 1 diabetes were followed prospectively for the first 10 years for the occurrence of infections and atopic diseases within The Environmental Determinants of Diabetes in the Young (TEDDY) study. A time‐discrete survival analysis was carried out to evaluate the association of self‐reported febrile and non‐febrile infections (respiratory, gastrointestinal, other, fever alone) and the risk of subsequent atopic eczema, allergic rhinitis, asthma, and allergies.

**Results:**

The rate of respiratory infections was positively associated with the subsequent risk of asthma (HR 1.30, 95% CI 1.25–1.35, *p* < .001). Febrile respiratory infections increased the risk of asthma more than non‐febrile ones (febrile respiratory infection HR 1.50, 95% CI 1.42–1.59, *p* < .001; non‐febrile respiratory infection HR 1.24, 95% CI 1.18–1.30, *p* < .001). In contrast, fever decreased the positive association of infections with the risk of other atopic diseases. Episodes of fever as the only reported symptom had a negative association particularly with the risk of atopic eczema (HR 0.53, 95% CI 0.39–0.71, *p* < .001).

**Conclusions:**

In a longitudinal cohort study, fever shows a clear impact on how infections associate with subsequent atopic diseases. Understanding the modifying role of fever on risk offers new possibilities to explore the mechanisms that mediate the effect of infections in the atopic process.

**Key message**

We propose for the first time in a large intercontinental prospective birth cohort with thorough follow‐up that fever has a major impact on subsequent risk of atopic diseases. Fever reinforces the positive association between respiratory infections and asthma, but—surprisingly—has the opposite effect for the association between infections and other atopic conditions. Fever as the sole symptom has a statistically significant inverse association with the incidence of subsequent atopic eczema and a borderline inverse association for allergic rhinitis. Our findings illustrate that fever seems to shield from other atopic diseases besides asthma. This finding calls for further studies and could have an impact on preventive protocols for atopic diseases.

## 1 INTRODUCTION

The incidence and burden of atopic diseases, such as asthma, allergic rhinitis, atopic eczema, and allergies, have grown high among children living in westernized countries^1^ due to reasons not fully known. The rapid increase suggests that, in addition to genetic predisposition, a modern lifestyle with a diminished exposure to diverse microbial environments could play a role.^2^, ^3^ According to the hygiene hypothesis proposed during the turn of the 80s to the 90s, this increase could be related to diminished microbial load in early childhood.^4^ Several studies since have provided indirect evidence supporting the hypothesis: e.g., having older siblings,^4^ growing up on a farm,^5^ early attendance in day care,^6^ vaginal delivery,^7^ diverse microbiome^8^ as well as many specific microbes^9^, ^10^, ^11^ have been shown to be inversely associated with atopic sensitization and/or atopic diseases.^12^ More studies are needed for a detailed list of environmental factors enhancing the atopy‐prone type 2 responses.^13^ It is not clear if it's the total burden of infections or just some types of infections, like orofecally transmitted infections^9^ or helminth parasites,^14^, ^15^ which might protect against atopic conditions. On the contrary, some studies haven't found an association between infections and atopic diseases.^16^ Furthermore, several studies indicate that some types of respiratory infections, especially wheezing respiratory illnesses linked to rhinovirus (related to both host and viral factors), and to some extent respiratory syncytial virus (RSV), can be risk factors for asthma.^17^, ^18^, ^19^, ^20^ Thus, the general picture of how infections impact atopic diseases is still blurred. There is a scarcity of prospective longitudinal follow‐up studies on a large scale with regular reporting of infections and details as to the presence of symptoms such as fever to examine the cumulative impacts over time on the risk of atopic disease.

The aim of this study is to shed light on the controversial role of infections in the development of atopic diseases in early life. Given what observations from prior studies have implicated, we hypothesized that a high rate of infections in early life may have heterogeneous effects on the risk of atopic diseases. We were especially interested in seeing how gastrointestinal infections could influence the risk of atopic diseases and expected to confirm a positive association between respiratory infections early in life and an increase in the risk of asthma. Moreover, this study sought to investigate if fever, a crude marker of infection severity and strong induction of immunomodulatory cytokines and chemokines, could modulate the association between infections and atopic disease. We had available diary‐based parent reports of various kinds of infections and atopic diseases from 3 months up to 10 years of age in The Environmental Determinants of Diabetes in the Young (TEDDY) Study to test these hypotheses. To our knowledge, this is one of the largest prospective birth cohort studies addressing the question.

## 2 METHODS

### 2.1 Setting and study population

**FIGURE 1:**
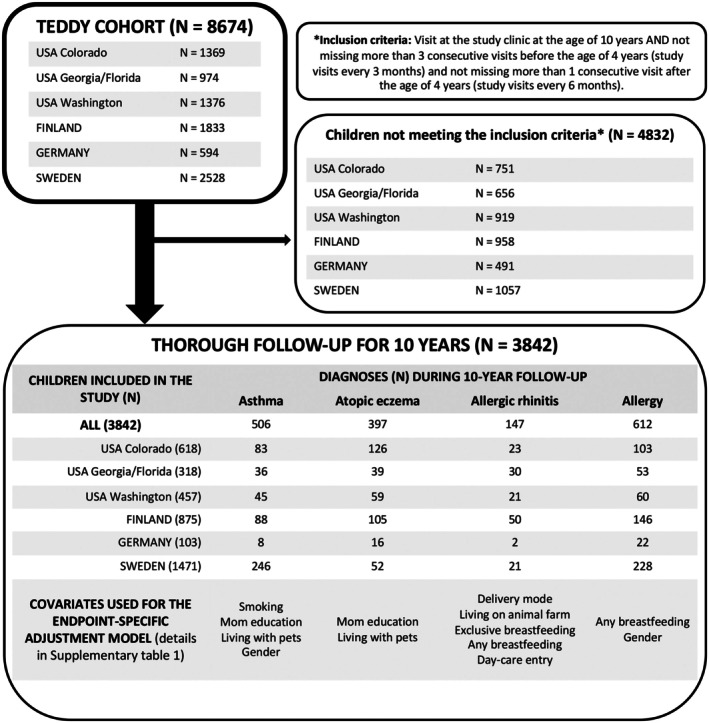
Descriptive statistics of the study flow with absolute numbers of children in each study center, outcome distributions, and key covariates.

The detailed description of the TEDDY study has been published previously.^21^, ^22^ In brief, the TEDDY study is a large multinational birth cohort study with six clinical research centers located in Finland, Germany, Sweden, and in the United States (Colorado, Georgia/Florida, and Washington). Population‐based HLA screening of new‐borns occurred between 2004 and 2010, after which 8674 children with HLA haplotypes conferring to an increased risk of type 1 diabetes (T1D) were enrolled in a follow‐up study until 15 years of age.^21^ Written informed consent was obtained from parents or primary caretakers for both genetic screening and participation in the prospective follow‐up. The still ongoing study was approved by local institutional review boards and is monitored by an external advisory board formed by the National Institute of Health. The study conforms to the standard of the Declaration of Helsinki. The first visit to the study clinic was scheduled at the age of 3–4 months, and follow‐up was every 3 months until the age of 4 years and every 6 months thereafter. Children developing islet autoimmunity stayed on the three‐month schedule. Between study visits, the parents prospectively recorded their child's infections using a TEDDY diary book. The current study utilizes the follow‐up data from study visits and the TEDDY diary book from 3 months until the age of 10 years. Children missing study visits for more than 3 consecutive times (interval of 3 months) before 4 years and, after that, more than 1 consecutive time (interval of 6 months) were excluded from the study. In addition, children not fulfilling a 10‐year follow‐up were excluded. Please see Figure 1 for a flow chart of the study population. At the end, 3842 children remained in our analyses. The participating research centers were represented as follows: 2449 from Europe (875 children from Finland, 103 from Germany, 1471 from Sweden) and 1393 from the United States (618 from Colorado, 318 from Georgia/Florida, and 457 from Washington).

### 2.2 Data on infectious episodes

A detailed description of the infectious disease report processing has been previously published.^23^ In brief, subjects' medical conditions—including infections—were recorded in the TEDDY diary book by parents. Parents filled in symptoms and/or diagnoses with the date of onset of each medical condition. At clinic visits, medical conditions reported in the TEDDY diary book were coded by trained study nurses according to the ICD‐10 classification of diseases. The ICD‐10 codes were collected in the TEDDY central database. Reports of fever were collected in the same database. For the present study, ICD‐10 codes for infectious diseases and reports of fever were extracted from the database. The extracted data were arranged into four groups: respiratory, gastrointestinal (GI), other known infectious episodes (such as chicken pox, herpes, urinary tract infection, bacterial skin disease, etc.), and episodes of fever alone (fever without any ICD‐10 code reported within a week prior to or after). Fever was determined as a temperature equal to or higher than 38°C in Europe or 101°F in the USA.

### 2.3 Data on atopic diseases

At each study visit, caregivers were asked for the presence of new atopic diseases and the date of the diagnosis made by a health care provider (“Since the last visit, has your child been diagnosed by a health care provider with any chronic illness or condition?”). This information of the parent‐reported diagnoses was coded into ICD‐10 codes by trained study nurses and added to the TEDDY central database. Diagnoses of asthma (J45, J45.0, J45.1, J45.8, J45.9), atopic eczema (L20, L20.0, L20.8, L20.9, L27.2), and allergic rhinitis/conjunctivitis (J30, J30.1, J30.2, J30.3, J30.4, H10.1) were used in this study. Caregivers also filled out information on different allergies in the TEDDY diary book, which were extracted to the TEDDY central database. The information was only accounted for if it was reported by the parents to be diagnosed by a health care provider (“Has a health care provider told you your child has this allergy?”). Allergy to pollens, furry animals, and common food allergies (cow's milk, egg, wheat, fish, peanut) were included in this study.

### 2.4 Statistical analysis

The discrete time survival (DTS) method was used as a statistical method in this cohort study. The statistical analyses were conducted using R version 4.2.2,^24^ utilizing the generalized linear model (glm) function with complementary log–log (cloglog) link function, allowing the results to be interpreted as hazard ratios (HR). A significance threshold of *p* = .05 was set.

Four different endpoints were used in the analyses: asthma, atopic eczema, allergic rhinitis, and allergy. Infections were categorized into four main classes: respiratory infections, GI infections, other infections, and fever alone. For statistical processing, the follow‐up time was divided into three‐month periods, during which the cumulative infection count was calculated and converted into a ratio of “infections per year.” The actual statistical analyses were performed using this ratio, and infections before the onset of the atopic disease were taken into account. HR indicates how a one‐unit change in the infection rate affects the risk of atopic disease at any given time point. Analysis was initially conducted separately for each infection class, and later infections were divided into febrile and non‐febrile infections. The Kaplan–Meier estimator was used to analyze the effect of at least one fever alone exposure before the age of 1 year on the risk of a child later receiving the diagnosis of atopic eczema.

## 3 RESULTS

### 3.1 Occurrence of atopic diseases

**FIGURE 2:**
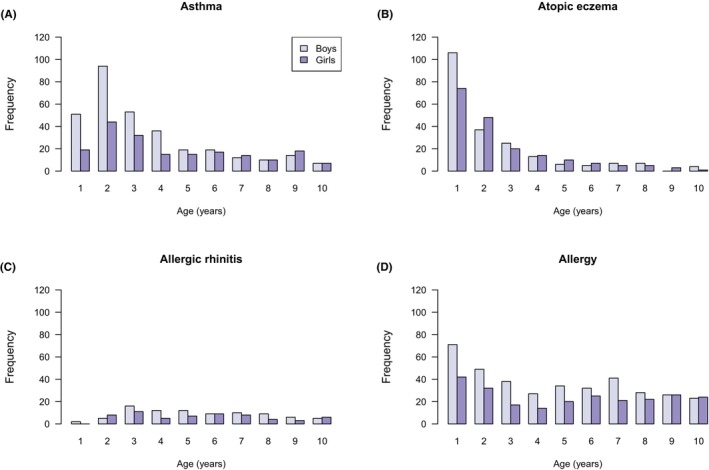
Frequency of atopic diseases according to the age at the time of the diagnosis. Girls are marked in a darker shade and boys in a lighter shade. On the x‐axis, the number 1 means the age between 0 and 1 years, the number 2 refers to the age between over 1 year up to 2 years, etc.

Of the 3842 children (1946 boys and 1896 girls) completing a comprehensive follow‐up until the age of 10 years, 1249 (37% of boys and 28% of girls, altogether 33%) had received a diagnosis of an atopic disease (asthma, atopic eczema, allergic rhinitis, or allergy) confirmed by a healthcare provider. The cumulative incidence of atopic diseases in the study population was as follows: 506 (13%) children with asthma (315 boys, 191 girls), 397 (10%) children with atopic eczema (210 boys, 187 girls), 147 (4%) children with allergic rhinitis (86 boys, 61 girls), and 612 (16%) children with an allergy diagnosis (369 boys, 243 girls). The distribution of endpoints can be seen in Figure 1. Figure 2 shows the onset of atopic diseases by age. Only one atopic disease was reported in 906 children, two in 283, three in 52, and all four in 8 children. The cumulative proportion of an atopic disease ranged from 29% to 41% in different research centres.

### 3.2 Occurrence of infections during the full follow‐up

**FIGURE 3:**
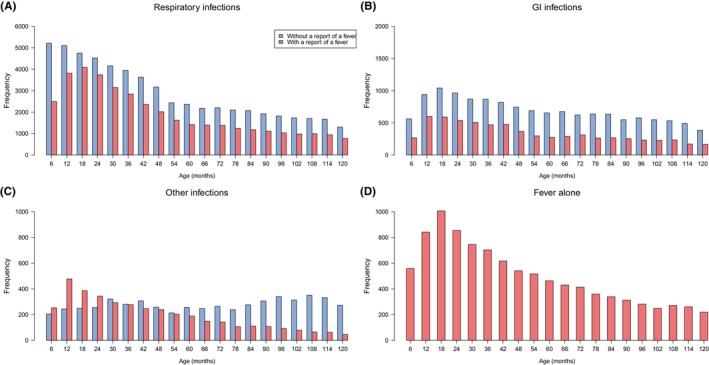
Frequency of infections (A respiratory, B gastrointestinal, C other infections and D fever alone without any other symptoms) by age (in months) in the full follow‐up cohort. Non‐febrile infections are shown in blue and febrile infections in red. Notice the difference in the scale of the y‐axis in different panels. Age 6 months on the x‐axis refers to the time between 0 and 6 months, age 12 to the time between over 6 months up to 12 months etc. GI, gastrointestinal.

The number of different infections during the 10 years full follow‐up was as follows (with fever in brackets): 96,573 (38,570) respiratory infections, 20,609 (6806) gastrointestinal infections, 9395 (3866) other infections, and 9984 fever alone infections. Infectious episodes peaked at 12–18 months (Figure 3).

### 3.3 Variables for adjustment

Variables for adjustment were selected by analyzing the effect of each potential covariate on different endpoints separately (asthma, allergic rhinitis, atopic eczema, allergy); please see Table S1. When the effect was statistically significant (*p* < .05) for the endpoint, the variable was included in the separate endpoint‐specific adjusted model for each of the atopic diseases. The plausible variables for adjustment were: gender (male/female), delivery (vaginal/other), child regularly spending time with a smoker (yes/no), HLA category, mother's education (high school or less/more than high school), living with animals/pets (yes/no), living on a farm with animals (yes/no), exclusive breastfeeding 3 months (yes/no), total/partial breastfeeding for at least 6 months (yes/no), day care entry (before vs. after the median age of 18 months), and siblings at birth (yes/no). The adjusted results are given in the main text. All the non‐adjusted and adjusted HR, 95% CI, and *p* values are listed in Table S2.

### 3.4 Infections and the risk of asthma

Respiratory infections (N/year) before the disease diagnosis were associated with the onset of asthma with a hazard ratio (HR) of 1.30 (95% CI 1.25–1.35, *p* < .001). Respiratory infections with fever were more strongly associated with the risk of asthma (HR 1.50, 95% CI 1.42–1.59, *p* < .001) than respiratory infections without fever (HR 1.24, 95% CI 1.18–1.30, *p* < .001). In addition, the group other infections showed a slight positive association with asthma: HR 1.27 (95% CI 1.05–1.53, *p* = .01). Gastrointestinal infections and episodes of fever alone had no association with asthma (HR 1.04, 95% CI 0.89–1.21, *p* = .36 and HR 1.05, 95% CI 0.89–1.25, *p* = .55).

### 3.5 Infections and the risk of atopic eczema

Respiratory infections with fever had a borderline inverse association with atopic eczema (HR 0.92, 95% CI 0.84–1.01, *p* = .076), while respiratory infections without fever surprisingly had the opposite effect (HR 1.09, 95% CI 1.03–1.15, *p* = .002). Gastrointestinal infections had no statistically significant association with atopic eczema (HR 1.06, 95% CI 0.90–1.26, *p* = .47). The group other infections had a slight positive association (HR 1.23, 95% CI 1.02–1.48, *p* = .03), and this positivity was stronger with other infections without fever (HR 1.34, 95% CI 1.07–1.68, *p* = .01). Finally, fever alone had a strong inverse association with atopic eczema: HR 0.53 (95% CI 0.39–0.71, *p* < .001).

**FIGURE 4:**
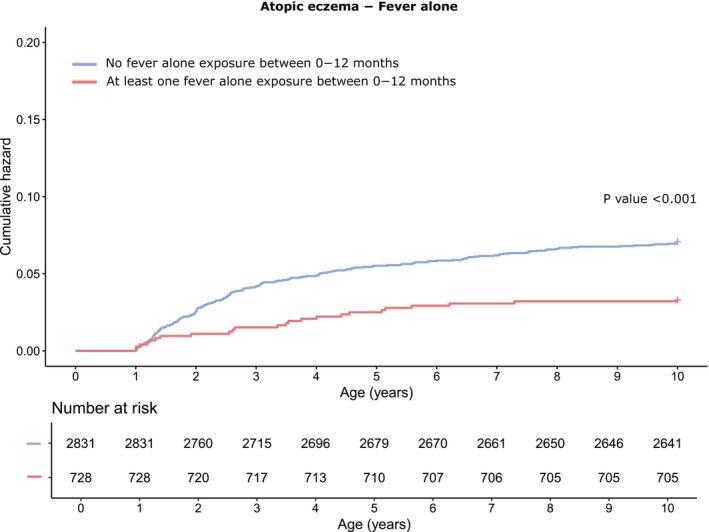
Cumulative hazard for atopic eczema after 1 year of age depending on whether or not a child had encountered a fever alone episode without any other symptoms during the first year of life. The figure depicts the Kaplan Meier curves for atopic eczema if a child had encountered at least one exposure of fever alone before the age of 1 year in red line and if a child had not encountered any fever alone episode during 0–1 years in blue line. Those children that had encountered fever alone infection stayed healthier in regard to atopic eczema during the 10‐year‐follow‐up. Children that received diagnosis for atopic eczema before the age of 1 year were not included in this analysis to make sure that the fever alone episode preceded the outcome.

The strong association of fever alone and atopic eczema was studied with survival analysis Kaplan–Meier estimate (Figure 4) to see if the timing of at least one fever alone exposure before the age of 12 months had an impact on the risk of a child getting the diagnosis of atopic eczema later on. Children having a fever alone exposure within 0–12 months compared with children without it had a significantly lower likelihood of getting a diagnosis of atopic eczema (*p* < .001).

### 3.6 Infections and the risk of allergic rhinitis

Respiratory infections and allergic rhinitis had a positive association (HR 1.32, 95% CI 1.23–1.43, *p* < .001). This was more clearly seen with respiratory infections without fever (HR 1.41, 95% CI 1.29–1.54, *p* < .001). Gastrointestinal infections again had no statistically significant association (HR 1.26, 95% CI 0.92–1.71, *p* = .15). For the group other infections, the HR was 1.61 (95% CI 1.16–2.25, *p* = .005) and, again, without fever the association was stronger HR 1.78 (95% CI 1.24–2.57, *p* = .002). For fever alone there was a trend, not statistically significant, for an inverse association with HR 0.72 (95% CI 0.45–1.14, *p* = .16).

### 3.7 Infections and the risk of allergies

Respiratory infections, especially without fever, and other infections without fever had a positive association with allergies HR 1.11 (95% CI 1.05–1.16, *p* < .001) and HR 1.41 (95% CI 1.11–1.80, *p* = .005), respectively. With all other infection categories with/without fever, there were no statistically significant associations with allergy.

### 3.8 Fever decreases the risk of atopic diseases other than asthma

**FIGURE 5:**
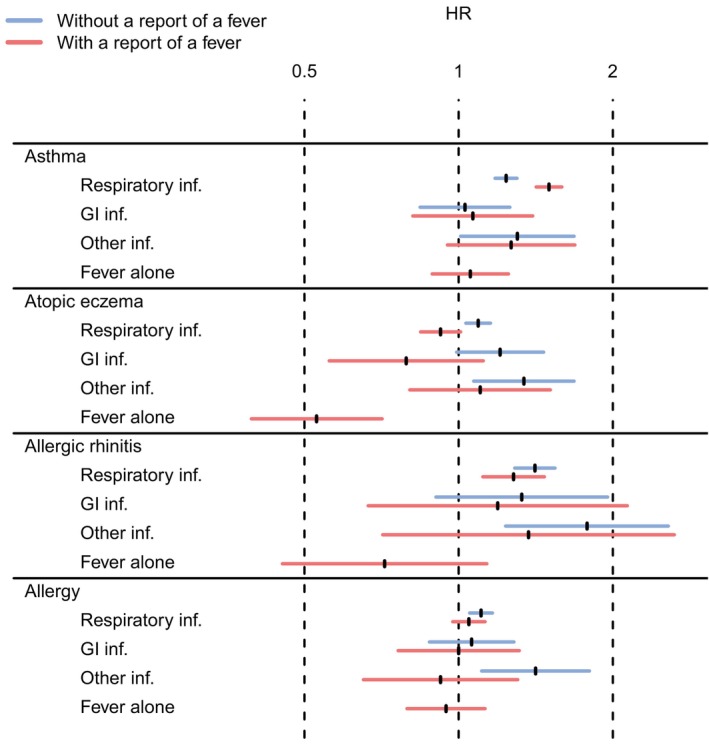
Hazard ratios (HR) for different atopic outcomes depending on different infection types with reported fever (red lines) and without reported fever (blue lines). Hazard ratios are calculated with time discrete survival analysis model explained in the text. Fever alone means infection episodes where parents reported fever but no other conventional symptoms of infection. Inf., infection; GI, gastrointestinal.

For other atopic diseases besides asthma, fever seems to reduce the possible risk association of the infections or even create an inverse association. This can be seen in Figure 5, which incorporates all the above‐mentioned endpoint‐specific adjusted hazard ratios of febrile and non‐febrile infections by groups into one figure.

### 3.9 Reproducibility of the results by study sites separately

The most significant associations seen in time discrete survival analyses were tested separately within the six study sites to observe if the results were universal or stemming mostly from one study site or continent. In all the study sites, the association of asthma and respiratory infections (both with and without fever separately) had hazard ratios ≥1. In addition, in all but one, the associations with asthma were stronger with respiratory infections with reported fever than without. Furthermore, in all of the study sites, the association of atopic eczema and fever alone had a hazard ratio below 1; however, in some (such as Finland and Germany), the hazard ratio was closely approaching 1. The above‐mentioned associations were all not statistically significant (*p* < .05) in every study site separately, as statistical power was lower especially for more seldomly acquired fever alone infections, but importantly, the similar tendency could be seen in each of them (data shown in Table S3).

## 4 DISCUSSION

We found that episodes of fever, particularly when occurring without other recorded symptoms of infection, showed a consistent inverse association especially with atopic eczema. We also confirm the previous findings of the positive association of respiratory infection episodes and the risk of developing asthma. This is reinforced with a simultaneous fever. Respiratory infections without fever had a statistically significant positive association with all the atopic diseases.

Fever has been shown to have a protective association with atopy and/or atopic diseases in some smaller cohorts previously.^12^, ^25^, ^26^, ^27^ In alignment with our results, the occurrence of fever during respiratory illnesses has been shown to be an important risk marker for childhood asthma.^28^ Infections are by far the leading cause of pediatric fever, also when no other symptoms are apparent.^29^ Other reasons for fever can be, for example, autoinflammatory recurrent fever, immune‐mediated diseases, malignancies, and genetic disorders, but these are extremely rare and should not play a role in the results.^30^ In addition, also immunizations can also cause a febrile episode. Immunizations and atopy/atopic diseases have been studied together without a clear‐cut association.^31^, ^32^ If the inverse association of fever seen in our study is caused by an unidentified infectious/other agent or fever as a phenomenon itself remains to be elucidated. However, the finding that various kinds of infections which presented with a fever showed a trend for a lower risk of atopic diseases (asthma excluded) than corresponding non‐febrile infections suggests that fever may reflect a certain type of host response to infections which regulates the risk of atopic diseases.

It has been shown in vitro that febrile temperatures can cause Th2 skewing of the effector T‐cell response through Notch signaling while also stimulating the counteracting IL‐12 production by dendritic cells.^33^ In addition to the innate immune cells, the adaptive immune cells can also sense febrile temperatures with complex consequences on the immunological balance, metabolic reprogramming, and production of cytokines such as IL‐1b, IL‐6, IL‐10, IL‐12, interferon gamma, and TNF‐alpha.^34^, ^35^, ^36^, ^37^, ^38^, ^39^, ^40^ Thus, fever can have the capacity to shape and train the balance of the immune system, leading to regulation of atopic response patterns.

When analyzing multiple comparisons, a Bonferroni correction factor can be used to adjust the statistical significance threshold to lower the risk of finding a significant association by chance. The Bonferroni correction factor (factor = 4 for different infection types) would yield an adjusted P value significance threshold of 0.0125. With this threshold, the statistical significance of the positive association between the group “other infections” and atopic eczema is lost. Other statistically significant associations presented here also exist also after the use of Bonferroni correction.

The strengths of this study are the large number of children prospectively followed using the same protocol, giving the study good statistical power. The study design lacks the challenges encountered in meta‐analyses in grouping together smaller studies that are not designed similarly. The parent‐reported atopic diseases had to be diagnosed by a health care professional to be regarded. The study has been conducted in six different research centers located in two continents, thereby increasing the generalizability of the results. And lastly, the prospective study design minimizes the possibility of recall bias and uncertainty of the timing of exposures related to outcomes.

This study also has its limitations: Our cohort consists of children with genetic susceptibility for T1D. The relationship between T1D and atopic diseases has been studied with controversial results. Some studies have shown that children with diabetic autoantibodies have fewer atopic diseases,^41^ while others have not found an association with atopic sensitization/disease and T1D.^42^, ^43^ The different HLA subgroups in this study did not show an association with atopic disease outcomes. However, due to the selection on specific HLA genotypes, the results may not be generalizable to the whole population. Limitation of selection bias can occur due to the inclusion criteria of precise follow‐up for the whole 10 years. Characteristics of these families with strong study adherence may associate with designated environmental factors and the risk of atopic disease. This must be considered when applying the results to the general population.

There are limitations in the definitions of the predictors and outcomes. We cannot ascertain that the diagnoses made by health care professionals were made according to the same diagnostic guidelines in all centres. We reckon that especially allergic rhinitis may have been underdiagnosed in this study as we believe some of the allergic rhinitis cases have been marked only in the allergy category (pollen/animal allergy). We also reckon that differentiating especially the repeated non‐febrile respiratory infections and allergic rhinitis can be difficult without verified microbial diagnostics and standardized allergy testing, leading to possible over‐ and underdiagnosis and skewed association between non‐febrile respiratory infections and allergic rhinitis. Finally, we could only include symptomatic infections and cannot draw any conclusions on the impact of asymptomatic infections and their role in the development of atopic diseases. Besides these, reporting of infections can be prone to variation due to differences between families in study compliance and vigilance in noticing symptoms. If the associations of infections and atopic diseases resulted only from the more diligent use of the TEDDY diary, we would not be expecting to see the inverse associations seen especially with fever.

Even though there was an inverse association of preceding fever episodes and many atopic diseases, and a positive association of preceding respiratory infections and atopic diseases, especially asthma, we cannot confirm the causality between the outcomes and the exposures. One study found patients with allergic rhinitis to have more numerous and prolonged respiratory infections than nonallergic subjects at adult age.^44^ There might be an intrinsic trait such as mucosal inflammation, impaired innate immunity, and a reduced Th1 response in allergic patients to predispose them to infections.^45^ Sometimes the actual symptoms of atopic disease and infection can overlap, as in respiratory infections and allergic rhinitis and, to some extent, asthma.

In conclusion, the present study suggests that fever, as a plausible specific host response pattern to infections, is associated with reduced risk of atopic diseases other than asthma. We show that the relationship of infections and atopic diseases must be studied separately for all the atopic diseases (asthma, atopic eczema, allergic rhinitis, and allergies) as they differ in the direction and magnitude of association. In addition, the presence of fever should be accounted for in studies considering the relationship of infections and atopic diseases. This observation opens new possibilities to study the mechanisms of how infections can shape the function of the immune system during childhood. A deep understanding of early‐life infectious episodes that impact the risk of atopic diseases is important for establishing good prevention and treatment strategies.

## AUTHOR CONTRIBUTIONS

**Tiina Palmu:** Conceptualization (equal); formal analysis (supporting); visualization (equal); writing – original draft (lead); writing – review and editing (equal). **Jussi Lehtonen:** Formal analysis (lead); visualization (equal); writing – original draft (supporting); writing – review and editing (equal). **Helena Elding Larsson:** Writing – review and editing (equal). **Kristian F. Lynch:** Writing – review and editing (equal). **Berglind Jonsdottir:** Writing – review and editing (equal). **Corrado Cilio:** Writing – review and editing (equal). **Desmond Schatz:** Writing – review and editing (equal). **Marian Rewers:** Writing – review and editing (equal). **Heikki Hyöty:** Conceptualization (equal); supervision (lead); writing – review and editing (equal). **Maria Lönnrot:** Conceptualization (equal); supervision (lead); visualization (equal); writing – original draft (supporting); writing – review and editing (equal).

## FUNDING INFORMATION

The study was supported by a grant from the Finnish Allergy Research Foundation, Finnish Dermatological Society and Tampere Tuberculosis Foundation. In addition, this project has received funding from the European Union's Horizon 2020 research and innovation programme under grant agreement No 874864 HEDIMED. The TEDDY Study is funded by U01 DK63829, U01 DK63861, U01 DK63821, U01 DK63865, U01 DK63863, U01 DK63836, U01 DK63790, UC4 DK63829, UC4 DK63861, UC4 DK63821, UC4 DK63865, UC4 DK63863, UC4 DK63836, UC4 DK95300, UC4 DK100238, UC4 DK106955, UC4 DK112243, UC4 DK117483, U01 DK124166, U01 DK128847, and Contract No. HHSN267200700014C from the National Institute of Diabetes and Digestive and Kidney Diseases (NIDDK), National Institute of Allergy and Infectious Diseases (NIAID), Eunice Kennedy Shriver National Institute of Child Health and Human Development (NICHD), National Institute of Environmental Health Sciences (NIEHS), Centers for Disease Control and Prevention (CDC), and Breakthrough T1D (formerly JDRF). This work is supported in part by the NIH/NCATS Clinical and Translational Science Awards to the University of Florida (UL1 TR000064) and the University of Colorado (UL1 TR002535). The content is solely the responsibility of the authors and does not necessarily represent the official views of the National Institutes of Health.

## CONFLICT OF INTEREST STATEMENT

The authors state that they have no conflict of interest.

## PEER REVIEW

The peer review history for this article is available at https://www.webofscience.com/api/gateway/wos/peer‐review/10.1111/pai.70209.

## Supporting information


Appendix S1.

Table S1.

Table S2.

Table S3.
